# Supplementary material for: Effects of Membrane and Biological Target on the Structural and Allosteric Properties of Recoverin: A Computational Approach
Source: Int J Mol Sci. 2019 Oct 10;20(20):5009. doi: 10.3390/ijms20205009 (PMC6829511; doi:10.3390/ijms20205009)
Supplement: Supplementary file 1 [file ijms-20-05009-s001.zip › ijms-610663 suppl for final/ijms-610663 suppl for final.pdf]

International Journal of *Molecular Sciences*

MDPI

Supplementary Materials

Effects of Membrane and Biological Target on the Structural and Allosteric Properties of Recoverin: A Computational Approach

Alberto Borsatto [1,2], Valerio Marino [1,3], Gianfranco Abrusci [2,4], Gianluca Lattanzi [2,4,*] and Daniele Dell'Orco [1,*]

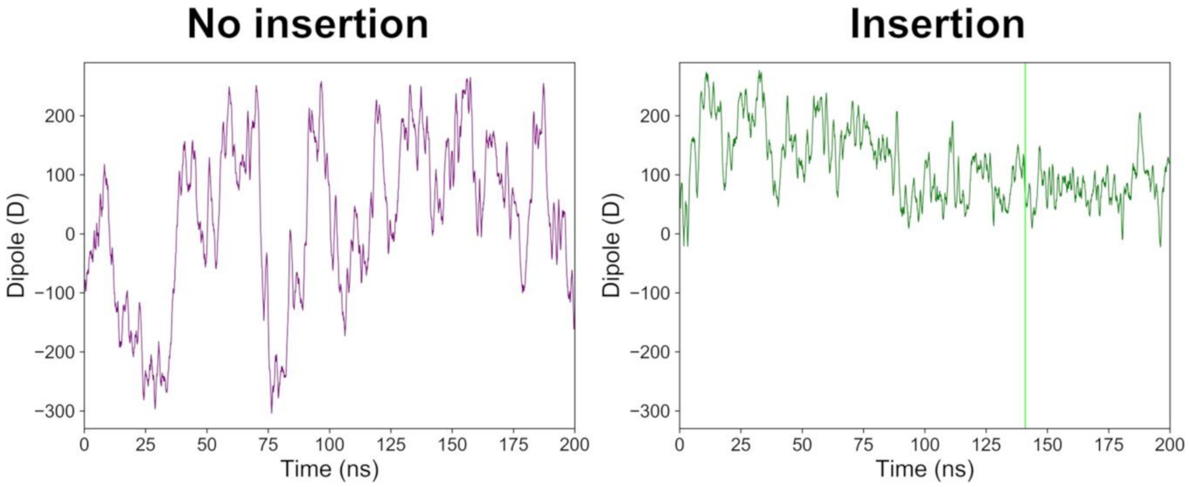

Figure S1. Time evolution of the dipole moment of Rec-R along Z axis over two 200 ns replicas where no myristoyl insertion was observed (left) and where myristoyl insertion occurred (right) in correspondence of the green line.

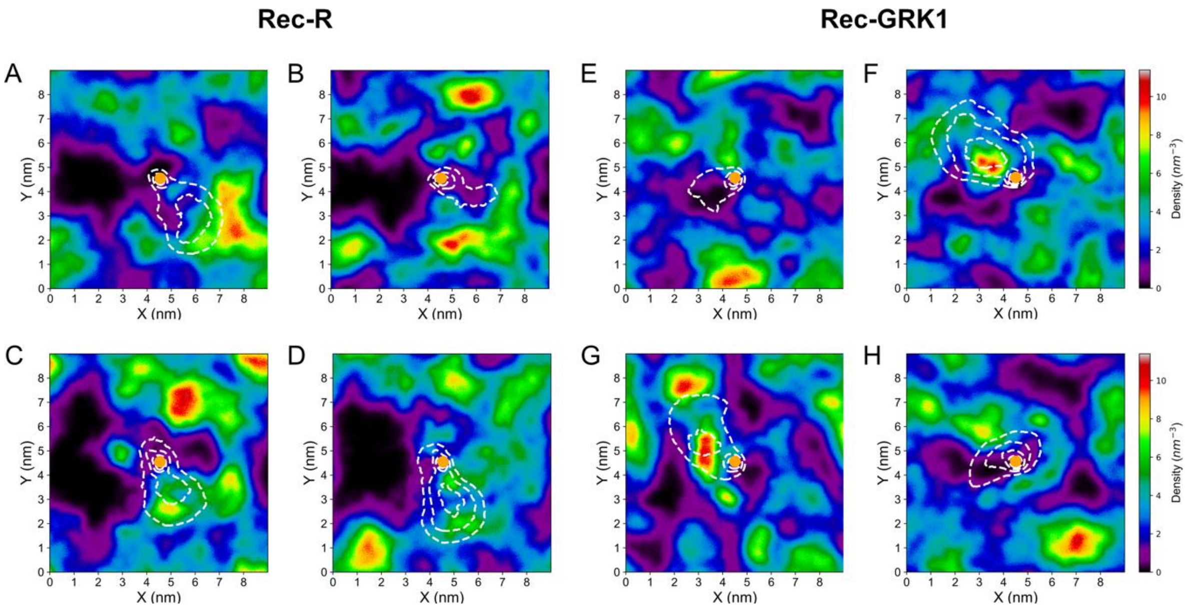

Figure S2. Density maps for DGPS upper membrane leaflet, calculated as the ratio between the number of atoms belonging to DGPS and the volume. The maps for Rec-R (A-D) and Rec-GRK1 (E-H) were generated analyzing all other four 200 ns membrane-anchored trajectories. Trajectories were centered on the myristoyl group, white dashed lines represent the contour of Rec density profiles of each replica projected onto the DGPS density maps.

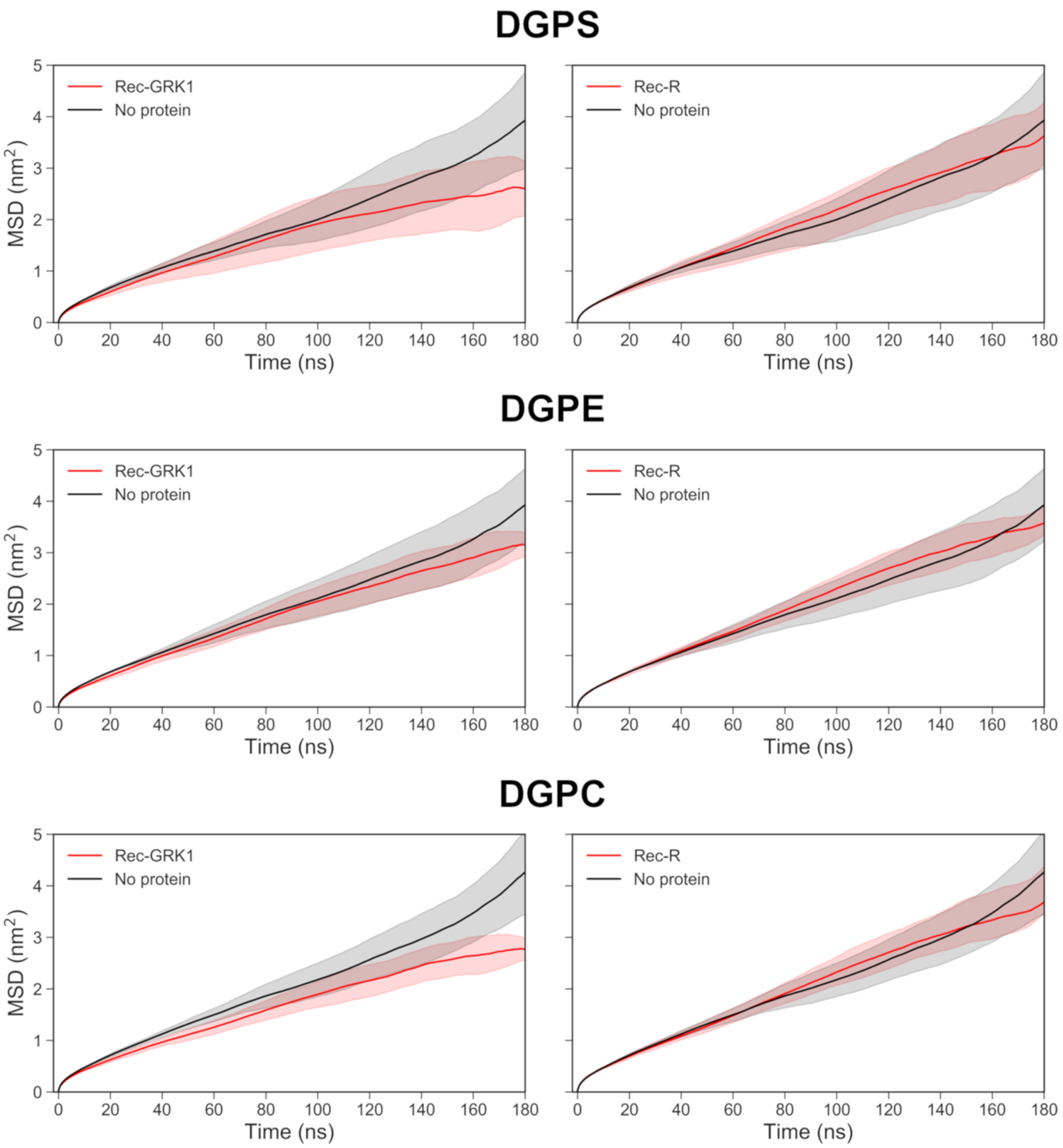

Figure S3. Mean square displacements (MSD) for the different lipid molecules in the membrane. The MSDs are calculated on 200 ns trajectories after protein insertion (red lines) or before protein insertion (black lines). Error bars (shaded areas) correspond to averages over various replicas. The time windows of 180 ns ensured proper statistics over the 200 ns trajectories.

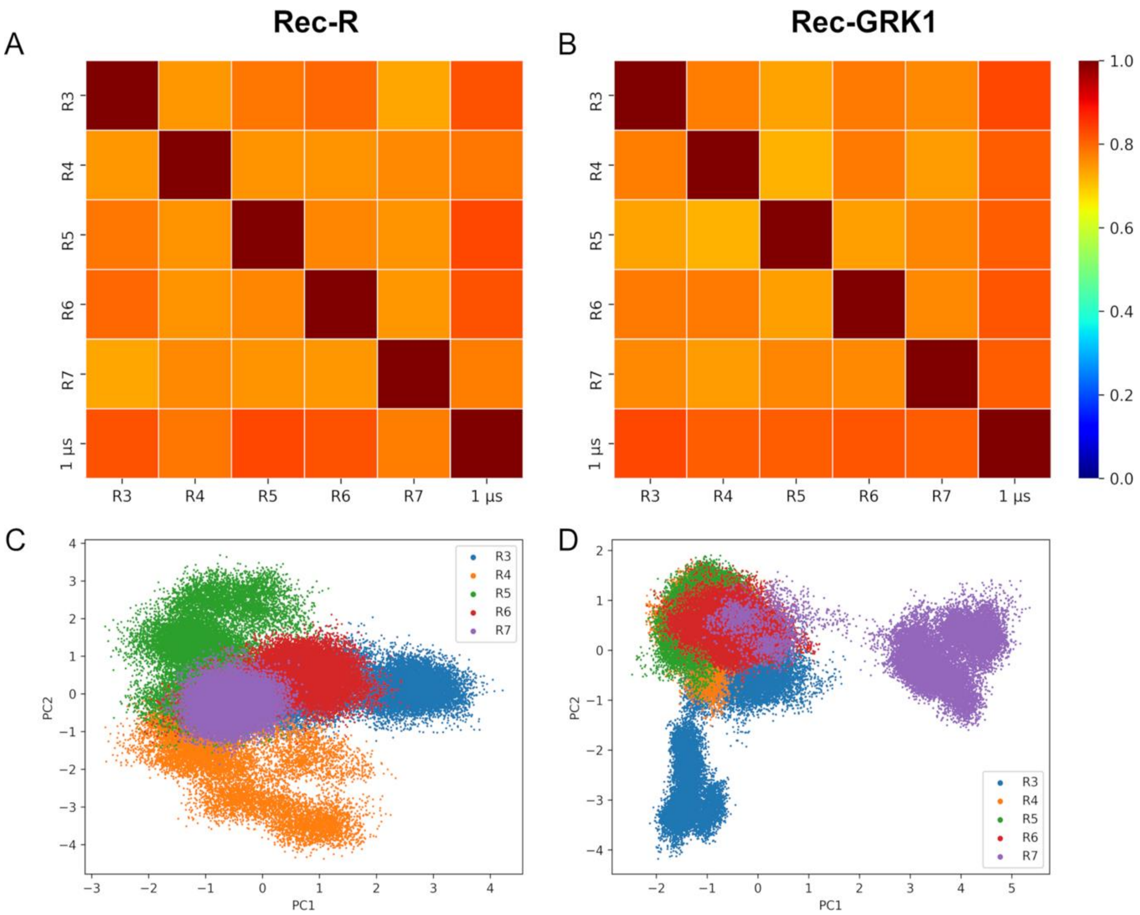

Figure S4. Root-Mean Square Inner Product (RMSIP) of the Essential Subspace constituted by the first 20 Principal Components of each of the five 200 ns MD simulation replicas (R3 to R7) and of the concatenated 1 µs trajectories of Rec-R (A) and Rec-GRK1 (B). Projections of the frames extracted from each of the five 200 ns MD simulation replicas (R3 to R7) along the first two Principal Components (PC1 and PC2) calculated on the concatenated 1 µs trajectories of Rec-R (C) and Rec-GRK1 (D).

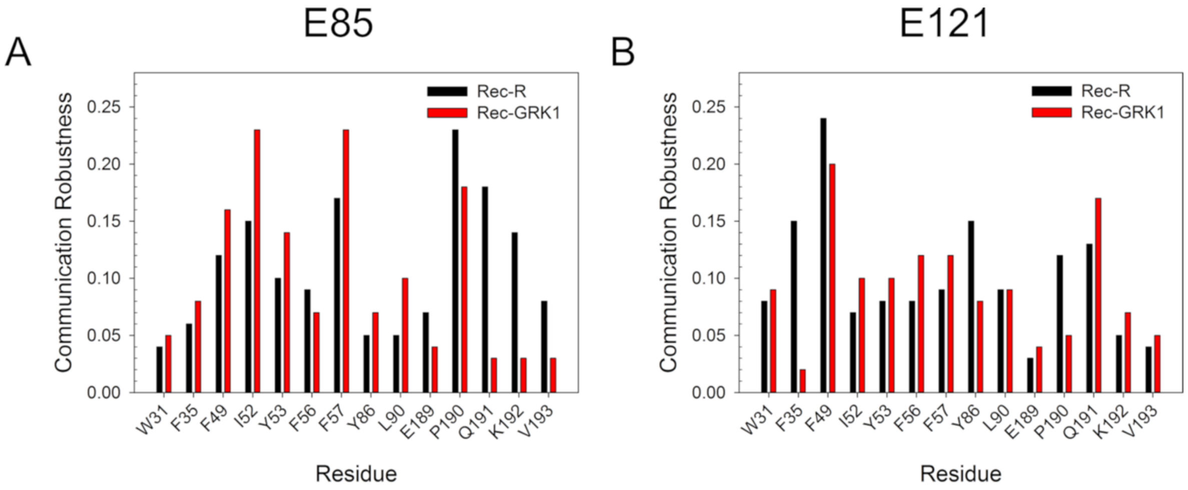

Figure S5. Intramolecular communication between Rec E85 (A) and E121 (B), representative residues of EF2 and EF3 respectively, and GRK1 interface residues. CR index is reported for all Rec interface residues of Rec-R (black) and Rec-GRK1 (red).

© 2019 by the authors. Submitted for possible open access publication under the terms and conditions of the Creative Commons Attribution (CC BY) license (http://creativecommons.org/licenses/by/4.0/).
